# Distinguishing the species of biomedical named entities for term identification

**DOI:** 10.1186/1471-2105-9-S11-S6

**Published:** 2008-11-19

**Authors:** Xinglong Wang, Michael Matthews

**Affiliations:** 1 National Centre for Text Mining, University of Manchester, 131 Princess Street, Manchester, M1 7DN, UK; 2 School of Informatics, University of Edinburgh, Informatics Forum, 10 Crichton Street, Edinburgh, EH8 9AB, UK; 3 The work described in this paper was carried out at School of Informatics, University of Edinburgh, UK

## Abstract

**Background:**

Term identification is the task of grounding ambiguous mentions of biomedical named entities in text to unique database identifiers. Previous work on term identification has focused on studying species-specific documents. However, full-length articles often describe entities across a number of species, in which case resolving the ambiguity of model organisms in entities is critical to achieving accurate term identification.

**Results:**

We developed and compared a number of rule-based and machine-learning based approaches to resolving species ambiguity in mentions of biomedical named entities, and demonstrated that a hybrid method achieved the best overall accuracy at 71.7%, as tested on the gold-standard ITI-TXM corpora. By utilising the species information predicted by the hybrid tagger, our rule-based term identification system was improved significantly by up to 11.6%.

**Conclusion:**

This paper shows that, in the context of identifying terms involving multiple model organisms, integration of an accurate species disambiguation system can significantly improve the performance of term identification systems.

## Background

The exponential growth of the amount of scientific literature in the fields of biomedicine and genomics has made it increasingly difficult for scientists to keep up with the state of the art. The TXM project [1], a three-year project which aims to produce software tools to aid curation of biomedical papers, targets this problem and exploits natural language processing (NLP) technology in an attempt to automatically extract enriched protein-protein interactions (EPPI) and tissue expressions (TE) from biomedical text.

A critical task in TXM is term identification (TI), the task of grounding mentions of biomedical named entities to identifiers in referent databases. TI can be seen as an intermediate task that builds on the previous component in an information extraction (IE) pipeline, i.e., named entity recognition (NER), and provides crucial information as input to the more complex module of relation extraction (RE). The structure of the IE pipeline resembles a typical curation process by human biologists. For example, when curating protein-protein interactions (PPIs), a curator would first mark up the protein mentions in text, and then identify the mentions by finding their unique identifiers from standard protein databases such as RefSeq [2], and finally curate pairs of IDs as PPIs.

TI is a matching and disambiguation process [3], and a primary source of ambiguity lies in the model organisms of the terms. In curation tasks, one often needs to deal with collections of articles that involve entities of a large variety of species. For example, our collection of articles from PubMed and PubMed Central involve over 100 model organisms. Also, it is often the case that more than one species appear in the same document, especially when the document is a full-length article. In our dataset, 74% of the articles concern more than one organism. In many standard databases, such as RefSeq and SwissProt, homolog proteins in different species, which often contain nearly identical synonym lists, are assigned distinct identifiers. This makes biomedical terms even more polysemous and hence species disambiguation becomes crucial to TI. For example, querying RefSeq with the protein mention *plk1 *resulted in 98 hits. By adding a species to the query, e.g. *mouse*, one can significantly reduce the number of results to two.

The most relevant work to ours are the *Gene Normalisation *(GN) tasks [4,5] in the BioCreAtIvE I & II workshops [6,7]. The data provided in the GN tasks, however, were species-specific, which means that the lexicons and datasets were concerned with single model organisms and thus species disambiguation was not required. A few participating systems, however, integrated a filter to rule out entities with erroneous species [8,9], which were reported to be helpful. Another difference between our task and the BioCreAtIvE GN ones is that we carry out TI on entity level while GN on document level.

It is worth mentioning that the protein-protein interaction task (IPS) in BioCreAtIvE II has taken into account species ambiguity. The IPS task resembles the work-flow of manual curation of PPIs in articles involving multiple species, and to accomplish the task, one would require a full pipeline of IE systems, including named entity recognition, term identification and relation extraction. The best result for IPS[10] was fairly low at 28.85% *F*1, which reflects the difficulty of the task. Some participants of IPS have reported (e.g., [11]) that resolving species ambiguity was one of the biggest challenges. Our analysis of the IPS training data revealed that the interacting proteins in this corpus belong to over 60 species, and only 56.27% of them are *human*.

As noted in previous work [10-14], determining the correct species for the protein mentions is a very important step towards TI. However, as far as we know, there has been little work in species disambiguation and in to what extent resolving species ambiguity at an entity level can help TI.

## Results and discussion

### Species disambiguation

The species tagger was developed on the ITI TXM corpora [15], which were produced as part of the TXM project [1]. We created two corpora in slightly different domains, EPPI and TE. The EPPI corpus consists of 217 full-text papers selected from PubMed and PubMed Central and domain experts annotated all documents for both protein entities and PPIs, as well as extra (enriched) information associated with the PPIs and normalisations of the proteins to publicly available ontologies. The TE corpus consists of 230 full-text papers, in which entities such as proteins, tissues, genes and mRNAcDNAs were identified, and a new tissue expression relation was marked up.

We used these corpora to develop a species tagging system. The biomedical entities in the data were manually assigned with standard database identifiers, where *genes *were assigned with EntrezGene IDs, and *proteins *and *mRNAcDNAs *with RefSeq IDs. Hence it was straightforward to obtain their species IDs through the mappings provided by EntrezGene and RefSeq. In more detail, proteins, protein complexes, genes and mRNAcDNAs in both EPPI and TE datasets were assigned with NCBI Taxonomy IDs (TaxIDs) [16], to denote their species. The EPPI and TE datasets have different distributions of species. For example, the entities in the EPPI training data belong to 118 species with *human *being the most frequent at 51.98%, and those in the TE training set are across 67 species and *mouse *was the most frequent at 44.67%.

To calculate the inter-annotator-agreement, about 40% of the documents were doubly annotated by different annotators. The averaged *F*1 scores of species annotation on the doubly annotated EPPI and TE datasets are 86.45% and 95.11%, respectively, indicating that human annotators have high agreement when assigning species to biomedical entities.

To assess how much species ambiguity accounts for the overall ambiguity in biomedical entities, we estimated the averaged *ambiguity rates *for the protein entities in the TXM datasets, without and with the species information.

Suppose there are *n *unique protein mentions in a dataset. First, we look up the RefSeq database by exact match with every unique protein mention *m*_*i*_, where *i *∈ {0..*n *- 1}, and for each *m*_*i *_we retrieve two lists of identifiers: *L*_*i *_and ${L^{\prime }}_{i}$, where *L*_*i *_consists of all identifiers and ${L^{\prime }}_{i}$ only contains the identifiers whose model organism matches the manually tagged species of the protein mention. The ambiguity rates without and with species are computed by $\frac{\sum_{i=0}^{n-1}\left|L_{i}\right|}{n}$ and $\frac{\sum_{i=0}^{n-1}\left|{L^{\prime }}_{i}\right|}{n}$, respectively. Table 1 shows the ambiguity rates on the EPPI and TE datasets.

**Table 1:** Ambiguity in protein names. Ambiguity in protein entities, with and without species information, in EPPI and TE datasets.

	Protein Cnt	ID Cnt	Ambiguity
EPPI	6,955	184,633	26.55
EPPI species	6,955	17,357	2.50
TE	8,539	103,016	12.06
TE species	8539	12,705	1.49

Using the ITI TXM corpora, we first devised a number of rule-based species disambiguation systems. It is intuitive that a species word that occurs near an entity (e.g., *"mouse p53"*) is a strong indicator of its species. To assess this intuition, we developed a set of rules using heuristics and the species words detected by a species word tagger (to be described later).

• *PreWd*: If the word preceding an entity is a species word, assign the species indicated by that word to the entity.

• *PreWd Sent*: If a species word occurs to the left of an entity and in the same sentence, assign the species indicated by that word to the entity.

• *Prefix*: If an entity has a species-indicating prefix, e.g., *mSos-1*, then tag the species to that entity.

• *Spread*: Spread the species of an entity *e *to all entities in the same document that have the same surface form with *e*. This rule must be used in conjunction with the other rules.

• *Majority Vote*: Count the species words in a document and assign as a weight to each species the proportion of all species words in the document that refer to the species. For example, if there are *N *species words in a document and *N*_*human *_are associated with *human*, the *human *species weight is calculated as $\frac{N_{human}}{N}$. Tag all entities in the document the species with the highest weight, defaulting to *human *in the case of a tie. This rule was used by default in the rule-based TI system, described later in this paper.

Table 2 shows the results of species tagging when the above rules were applied. As we can see, the precision of the systems that rely solely on the previous species words or prefixes is very good but the recall is low. The system that looks at the previous species word in the same sentence does better as measured by *F*1. In addition, spreading the species improves both systems but the overall results are still not satisfactory.

**Table 2:** Results (%) of the rule-based species tagger

	EPPI devtest			TE devtest		
	
	P	R	F1	P	R	F1
PreWd	81.88	1.87	3.65	91.49	1.63	3.21
PreWd + Spread	63.85	14.17	23.19	77.84	17.97	29.20
PreWd Sent	60.79	5.16	9.52	56.16	7.76	13.64
PreWd Sent + Spread	39.74	50.54	44.49	31.71	46.68	37.76
Prefix	98.98	3.07	5.96	77.93	2.97	5.72
PreWd + Prefix	91.95	4.95	9.40	82.27	4.62	8.75
PreWd + Prefix + Spread	68.46	17.49	27.87	77.77	21.26	33.39
Majority Vote	44.10	44.10	44.10	49.87	49.87	49.87

It is slightly counter-intuitive that using a rule such as '*PreWd*' did not achieve perfect precision. Closer inspection revealed that most of the false positives were due to a few problematic guidelines in the annotation process. For example,

• "*The amounts of human and mouse CD200R *...", where 'CD200R' was tagged as *mouse (10090) *by the system but the gold-standard answer was *human (9606)*. This was due to the fact that the annotation tool was not able to assign multiple correct species to a single entity.

• "... *wheat *e*IFiso4G *...", where 'eIFiso4G' was tagged as *wheat (4565) *but the annotator thought it was *Triticum (4564)*. In this case, TaxID 4565 is a species under genus 4564, and arguably is also a correct answer. Other similar cases include Xenopus vs. Xenopus tropicalis, and Rattus vs. Rattus norvegicus, etc. This is the main cause for the false positives as our system always predicts species instead of genus or TaxIDs of any other ranks, which the annotators occasionally employed.

Furthermore, we split the EPPI and TE datasets into training and development test (devtest) sets and developed a machine-learning (ML) based species tagger. Using the training splits, we trained a maximum entropy classifier [17] on a number of features such as contextual words and nearly species words, which will be detailed later.

The results of the ML species tagger are shown in Table 3. We measure the performance in accuracy instead of *F*1 because the ML based tagger assigns a species tag to *every *entity occurrence, and therefore precision is equal to recall. We tested four models on the devtest portions of the EPPI and TE corpora:

**Table 3:** Results (%) of the machine-learning and hybrid species taggers. Accuracy (%) of the machine-learning based species tagger and the hybrid species tagger as tested on the EPPI and TE devtest datasets. An 'Overall' score is the micro-average of a system's accuracy on both datasets.

	BL	EPPI Model	TE Model	Combined Model	EPPI Model+Rules	TE Model+Rules	Combined Model+Rules
EPPI devtest	60.56	73.03	58.67	72.28	**74.24**	59.67	73.77
TE devtest	30.22	67.15	69.82	67.20	67.53	**70.14**	67.47
Overall	48.88	70.77	62.96	70.33	**71.66**	63.70	71.34

• *BL*: a baseline system, which tags the devtest instances using the most frequent species occurring in the corresponding training dataset. For example, *human *is the most frequent species in the EPPI training data, and therefore all entities in the EPPI devtest dataset were tagged with *human*.

• EPPI *Model*: obtained by training the maxent classifier on the EPPI training data.

• TE *Model*: obtained by training the maxent classifier on the TE training data.

• *Combined Model*: obtained by training the maxent classifier on a joint dataset consisting of both the EPPI and TE training corpora.

Finally, we devised a hybrid species tagging system. As we have shown, the rules '*PreWd*' and '*Prefix*' achieved very good precision but low recall, which suggests that when these rules were applicable, it is highly likely that they would get the correct species. Based on this observation, we combined the ML approach and the rule-based approach in such a way that the rules '*PreWd*' and '*Prefix*' were applied on top of ML and to override predictions made by ML. The hybrid systems were tested on the same datasets and the results are shown in the right 3 columns in Table 3. We performed significance tests on the results in Table 3. First, a Friedman test was used to determine whether the 7 sets of results were significantly different, and then pairwise Wilcoxon Signed Rank tests were employed to tell whether any system performed significantly better than others. On both datasets, the 6 machine-learning models significantly outperformed the baseline (*p <*0.01). On EPPI devtest dataset, the EPPI models (with or without rules) and the Combined Models outperformed the TE models (*p <*0.05), while on TE dataset, the TE models and the Combined Models outperformed the EPPI models (*p <*0.05). Applying the post filtering rules did not significantly improve the ML models, although it appears that adding the rules consistently increased the accuracy.

### Term identification with species disambiguation

#### Experiments on the ITI TXM corpora

To identify whether species disambiguation can improve performance of TI, we ran the TI system on the EPPI and TE datasets in the ITI TXM corpora. We tested the TI systems with or without help from a number of species tagging systems, including:

• *Baseline*: Run TI without species tags. Note that the TI system already integrated a basic species tagging system that uses the *Majority Vote *rule. Thus this is a fairly high 'baseline'.

• *Gold Species*: Run TI with manually tagged species. This is the upper-bound performance.

• *Rule*: Run TI with species predicted by the rule-based species tagger using rules "PreWd" and "Prefix".

• *ML*(*human/mouse*): Run TI with the species that occurs most frequently in the training datasets (i.e., *human *for EPPI and *mouse *for TE).

• *ML*(EPPI): Run TI with species predicted by the ML tagger trained on the EPPI training dataset.

• *ML*(EPPI)+*Rule*: Run TI with species predicted by the hybrid system using both ML(EPPI) and the rules.

• *ML*(TE): Run TI with species predicted by the ML tagger trained on the TE training dataset.

• *ML*(TE)+*Rule*: Run TI with species predicted by the hybrid system using both ML(TE) and the rules.

• *ML*(EPPI+TE): Run TI with species predicted by the ML tagger trained on both EPPI and TE training data.

• *ML*(EPPI+TE)+*Rule*: Run TI with species predicted by the hybrid system using both ML(EPPI+TE) and the rules.

We score the systems using *t*op n precision, where *n *∈ {1, 5, 10, 15, 20}. The argument for this evaluation scheme is that if a TI system is not good enough in predicting a single identifier correctly, a 'bag' of IDs with the correct answer included would also be helpful. The 'Avg. Rank' field denotes the averaged position where the correct answer lies in, and the lower the value is, the better the TI system performs. For example, a TI system with an 'Avg. Rank' of 1 would be ideal, as it would always return the correct ID at the top of the list. Note that in the TE data, not only protein entities, but also genes, mRNAcDNA, and GOMOPs were tagged, where a GOMOP denotes an entity being either a gene, or an mRNAcDNA, or a protein.

As shown in Tables 4 and 5, on both datasets, using the gold standard species much improved accuracy of TI (e.g., 19.2% on EPPI data). Also, automatically predicted species tags were proven to be helpful. On the EPPI data, the *ML*(EPPI)+*Rule *outperformed other systems. Note that the species distribution in the devtest dataset is strongly biased to *human*, which explains why the *ML*(*human*) system performed nearly as well. However, defaulting to *human *was not guaranteed to succeed because one would not be able to know the prior species in a collection of unseen documents. Indeed, on the TE data, the system *ML*(*mouse*), which uses *mouse *as default, yielded poor results.

**Table 4:** Results (%) of TI on the EPPI dataset. All figures, except 'Avg. Rank', are percentages. This evaluation was carried out on protein entities only.

Method	Prec@1	Prec@5	Prec@10	Prec@15	Prec@20	Avg. Rank
Baseline	54.31	73.45	76.44	77.90	78.51	5.82
Gold Species	73.52	79.36	80.75	80.75	80.99	1.62

Rule	54.99	73.72	76.45	77.91	78.52	5.79
ML(human)	65.66	76.36	78.82	79.78	80.03	2.58
ML(EPPI)	65.24	76.82	79.01	79.93	80.29	2.39
ML(EPPI)+Rule	**65.88**	**77.09**	**79.04**	**79.94**	80.30	**2.36**
ML(TE)	55.87	75.14	78.69	79.85	80.30	2.86
ML(TE)+Rule	56.54	75.47	78.70	79.86	80.31	2.83
ML(EPPI+TE)	64.55	76.48	78.53	79.83	80.38	2.49
ML(EPPI+TE)+Rule	65.03	76.62	78.55	79.84	**80.39**	2.46

**Table 5:** Results (%) of TI on the TE dataset. All figures, except 'Avg. Rank', are percentages. There are four entity types in the TE data, i.e., protein, gene, mRNAcDNA and GOMOP. The evaluation was carried out on all entity types.

Method	Prec@1	Prec@5	Prec@10	Prec@15	Prec@20	Avg. Rank
Baseline	63.24	76.20	77.30	77.94	78.25	1.72
Gold Species	71.82	78.03	78.34	78.40	78.41	1.29

Rule	63.45	76.21	77.30	**77.95**	**78.25**	1.72
ML(mouse)	58.76	75.40	77.25	77.92	78.24	1.90
ML(EPPI)	66.59	76.53	77.23	77.76	78.12	1.68
ML(EPPI)+Rule	**66.85**	**76.54**	77.24	77.76	78.12	**1.67**
ML(TE)	66.12	76.25	77.32	77.81	78.11	1.70
ML(TE)+Rule	66.37	76.25	**77.32**	77.81	78.11	1.70
ML(EPPI+TE)	65.78	76.14	77.28	77.84	78.12	1.71
ML(EPPI+TE)+Rule	66.03	76.14	77.29	77.84	78.12	1.70

#### Experiments on BioCreAtIvE data

To assess the portability of the species tagging approaches, an "artificial" dataset was created by joining the species-specific datasets from BioCreAtIvE 1 & 2 GN tasks to form a corpus consisting of four species. In detail, four datasets were taken, three from BioCreAtIvE 1 task 1B (i.e., fly, mouse and yeast) and one from BioCreAtIvE 2 task GN (i.e., human). Assuming genes in each dataset are species-specific, we can train/test ML models for species disambiguation and apply them to help TI. This task is more difficult than the original BioCreAtIvE GN tasks due to the additional ambiguity caused by multiple model organisms. Note that the above assumption is not strictly true because each dataset may contain genes of other species, and it would be hard to assess how true it is as abstracts in the BioCreAtIvE GN datasets are not normalised to an entity level.

We first carried out experiments on species disambiguation. In addition to the TXM (i.e., the system uses ML(EPPI+TE)+Rule model) and the *Majority Vote *taggers, we trained the species tagger on a dataset comprising of the devtest sets from the BioCreAtIvE I & II GN tasks. In more detail, we first pre-processed the dataset and marked up gene entities with an NER system [11,18], which was trained on BioCreAtIvE II GM training and test datasets. The entities were tagged with the species as indicated by the source dataset where they were drawn from, which were used as the 'gold' species. Using the same algorithm and feature set as described previously, a *BC model *was trained. As shown in Table 6, except on *human*, the TXM model yielded very disappointing results, whereas the BC model did well overall. This was because the TXM model was trained on a dataset where *fly *and *yeast *entities occur rarely with only 2% and 5% of the training instances belonging to these species, respectively, which again revealed the influence of the bias introduced in the training material to the ML models.

**Table 6:** Results (%) of species tagging on the BioCrAtIvE joint dataset. Accuracy (%) of the species disambiguation systems as tested on the BioCreAtIvE I & II test data. The 'BC model' was trained on the BioCreAtIvE devtest data, the 'TXM model' was trained on the TXMEPPI and TE training data, and the '*Majority Vote*' was the default species tagging system in the TI system.

	human	fly	mouse	yeast
Majority Vote	82.35	78.43	71.69	85.12
BC model	70.23	89.24	75.41	87.64
TXM model	93.35	3.27	31.89	3.49

Using the species disambiguation models, we carried out TI experiments, using the same procedure as we did on the TXM data. The results were obtained using the official BioCreAtIvE GN scorers and are presented in Table 7. Performance of TI assisted by all three species taggers were much behind that of TI using the gold-standard species, which shows species-tagging can potentially enhance TI performance and there is much room for improving the species disambiguation systems. On the other hand, it was disappointing that the '*Majority Vote*' system, which did not use any external species tagger, achieved the best results, while TI with the 'BC model' tagger yielded slightly worse results and the TXM model performed poorly.

**Table 7:** Results (%) of TI on the BioCrAtIvE joint dataset. Performance of TI with or without the automatically predicted species on the joint BioCreAtIvE GN test dataset.

System	Precision	Recall	*F*1
Gold	70.1	63.3	66.5
Majority Vote	46.7	56.3	51.0
TXM model	37.8	46.5	41.7
BC model	45.8	56.1	50.4

One possible reason that the '*Majority Vote*' tagger yielded reasonably good result on the BioCreAtIvE dataset, but unsatisfactory result on the TXM datasets was due to the difference in document length in the two corpora: the BioCreAtIvE corpus is comprised of abstracts and the TXM corpora consist of only full-length articles. In abstracts, authors are inclined to only talk about the main biomedical entities described in the paper, whereas in full articles, they tend to describe a larger number of entities, possibly in multiple species, for the purposes of describing related work or comparison. Recall that the '*Majority Vote*' rule outputs the species indicated by the majority of the species words, which would obviously perform better on abstracts, where more likely only one species is described, than on full-length articles. Table 8 shows the number of species per document in the TXM data, where most documents (i.e., 74%) involve more than one species, in which cases the '*Majority Vote*' would not be able to take obvious advantage.

**Table 8:** # of species per document in the TXM data

# Species	# of Docs	% of Docs
1	96	26.20
2	121	32.79
3+	153	41.19

## Conclusion

We have presented a range of solutions to the task of species disambiguation and evaluated their performance on the ITI TXM corpus, and on a joint dataset from BioCreAtIvE I & II GN tasks. We showed that rule-based species tagging systems that exploit heuristics, such as previous species words or species prefix, can achieve very high precision but low recall. ML species taggers, on the other hand, can achieve good overall performance, under the condition that the species distributions in training and test datasets are not too distant. Our best performing species tagger is a hybrid system that first uses ML to predict species and then applies certain rules to correct errors.

We also performed TI experiments with help from the species tags assigned by human annotators, or predicted by the automatic species taggers. On all datasets, the gold-standard species tags much improved TI performance: 19.21% on the EPPI devtest set, 8.59% on the TE devtest set, and 23.4% on the BioCreAtIvE GN test datasets, which clearly shows that species information is indeed very important for TI. On the EPPI and TE datasets, the species predicted by the best-performing hybrid system improved TI by 11.57% and 3.61%, respectively. On the combined dataset from BioCreAtIvE GN tasks, however, it did not work as well as expected.

## Methods

### Detecting species words

Words referring to species, such as *human*, are important indicators of the species of the nearby entities. We developed a rule-based program that detects *species words*, which were used to help the species identification systems.

The species word tagger is a lexical look-up component which applies to tokenised text and marks content words such as *human*, *murine *and *D. melanogaster *with their corresponding species TaxIDs. In addition, rules written in an *lxtransduce *grammar [19] developed at the LTG group at Edinburgh University are used to identify species prefixes (e.g., 'h' for *human*, 'm' for *mouse*). For example, the term *mSos-1 *would be assigned with a TaxID for *mouse*. Note that a species "word" may contain several words, for example, "E. coli". Please see [20] for more details on the species word tagger.

### Machine learning based species tagging

We trained a maximum entropy classifier [17] on the following set of features, with respect to each entity occurrence. The parameter *n *was empirically developed using the training datasets.

• *leftContext *The *n *word lemmas to the left of the entity, without position (*n *= 200).

• *rightContext *The *n *word lemmas to the right of the entity, without position (*n *= 200).

• *leftSpeciesIDs *The *n *species IDs, located to the left of the entity and assigned by the species word tagger (*n *= 5).

• *rightSpeciesIDs *The *n *species IDs, located to the right of the entity and assigned by the species word tagger (*n *= 5).

• *leftNouns *The *n *nouns to the left of the entity (with order and *n *= 2). This feature attempts to capture cases where a noun preceding an entity indicates species, e.g., *mouse protein p53*.

• *leftAdjs *The *n *adjectives to the left of the entity (with order and *n *= 2). This feature intends to capture cases where an adjective preceding an entity indicates species, e.g., *murine protein p53*.

• *leftSpeciesWords *The *n *species word forms, identified by the species word tagger, located to the left of the entity (*n *= 5).

• *rightSpeciesWords *The *n *species word forms, identified by the species word tagger, located to the right of the entity (*n *= 5).

• *firstLetter *The first character of the entity itself. Sometimes the first letters of entities indicate their species, e.g., *hP53*.

• *documentSpeciesIDs *All species IDs that occur in the article in question.

• *useStopWords *If this feature is switched on then filter out the words that appear in a pre-compiled stop-word list from the above features. The list consists of frequent common English words such as prepositions (e.g., *in*).

• *useStopPattern *If this feature is switched on then filter out the words consisting only of digits and punctuation characters.

### The TI system

The TI system is composed of a matcher which determines a list of candidate identifiers and a ranker that assigns a confidence value to each identifier that is used to rank the candidates in order with the most likely identifiers occurring first. The matcher is based largely on the rule-based system described in [3], but has been put into a more flexible framework that allows for defining and customising the rules in a configuration file. In addition, the system has been expanded to perform TI on additional entity types. The rules for each entity were developed using the training data and a visualisation system that compared the synonym list for the target identifiers with the actual entity mentions and provided visual feedback on the true positives and false positives resulting from candidate rules sets. Examples of some of the rules that can be incorporated into the system are listed below. A confidence value is assigned to each of the rules using heuristics and passed to the ranking system.

1. *LowerCase*: Convert the entity mention to lowercase and look up the result in a lower case version of the entity term database.

2. *Norm*: Normalise the mention and look up the result in a normalised version of the term database, where normalising a string involves converting Greek characters to English (e.g., *α *→ alpha), converting to lowercase, changing sequential indicators to integer numerals (e.g., *i*, *a*, *alpha *→ 1, etc.) and removing all spaces and punctuation. For example, *rab1*, *rab-1*, *rabα*, *rab I *are all normalised to *rab1*.

3. *Prefix*: Add and/or remove a set of prefixes from the entity mention and look up the result in the entity term database. The actual prefixes and whether to add or remove them are specified in the configuration file.

4. *Suffix*: Add and/or remove a set of suffixes from the entity mention and look up the result in the entity term database. The actual suffixes and whether to add or remove them are specified in the configuration file.

5. *Porter*: Compute the Porter stem of the entity mention and looked up the synonym in a Porter stemmed version of the entity term database.

The ranking system currently works by defining a set of confidence indicators for each entity, computing the confidence for each indicator and then multiplying each individual confidence together to determine the overall identifier confidence. The following indicators are currently used by the system.

1. *Match*: The confidence as determined by the matcher.

2. *Species*: The confidence that the species of the identifier is the correct species.

3. *Reference Count*: Based on the number of literature references databases associated with each identifier, obtained from EntrezGene and RefSeq. The higher the reference count, the higher the confidence.

4. *Primary Name*: Based on a determination that the entity mention is the primary name for the identifier. This is based both on a name provided by the lexicon and a name derived from the synonym list.

Among these, one of the most critical indicators is the species confidence. By default, this confidence is set to the weight assigned to the species by the *Majority Vote *tagger. When the species of an entity is tagged by an external species tagger or by human annotators, the default confidence can be overridden. This setting allows us to integrate automatic species taggers, such as the ones described in the previous section, for achieving better TI performance. For example, suppose we want to employ the *Hybrid *species tagger. To compute the species confidence, first the hybrid tagger is used to predict the most likely species and the *Majority Vote *tagger is run at the same time. If the species of an identifier matches the species assigned by the hybrid tagger, the species confidence is set to the weight generated by the hybrid tagger. Otherwise, the confidence is set to the weight generated by the *Majority Vote *tagger.

## Competing interests

This work was funded by ITI Life Sciences, Scotland, whose mission is to explore commercialisation of promising technologies in the life sciences.

## Authors' contributions

XW developed the species disambiguation systems and carried out experimentation and evaluation on integrating the species information to the rule-based term identification system. MM developed the rule-based term identification system. Both authors contributed to preparation of this manuscript.
